# Supplementary material for: Comparison of cardiac output estimates obtained from the Antares oscillometric pulse wave analysis algorithm and from Doppler transthoracic echocardiography
Source: PLoS One. 2024 May 13;19(5):e0302793. doi: 10.1371/journal.pone.0302793 (PMC11090340; doi:10.1371/journal.pone.0302793)
Supplement: Attachment — Submitted filename: Response to Reviewers.docx [file pone.0302793.s002.docx]

|  | Dr. Alexander Stäuber  Leipzig University  Movement and Training Science  Jahnallee 59  04109 Leipzig  Germany  e-mail: alexander.staeuber@uni-leipzig.de |
| --- | --- |

Academic Editor

*PLOS ONE*

March 20, 2024

Dear Prof. Dr. Giacomo Pucci:

We hereby resubmit our major revised manuscript entitled "Comparison of cardiac output estimates obtained from the Antares oscillometric pulse wave analysis algorithm and from Doppler transthoracic echocardiography” (ID: PONE-D-24-03078).

We sincerely appreciate your valuable advice and the insightful suggestions and comments provided by the reviewers aimed at enhancing the quality of our manuscript to align with PLOS ONE’s publication standards. We have thoroughly considered all the points raised by both you and the reviewer. To ensure clarity and transparency, we have addressed each comment individually by restating it and offering our response to the best of our ability. All required modifications have been clearly highlighted in the manuscript file ('Revised Manuscript with Track Changes'). Thank you once again for your constructive feedback and guidance throughout this process, which helped us to improve the quality of the paper clearly.

Thank you for your consideration!

Sincerely,

Dr. Alexander Stäuber

**Academic Editor | Journal requirements**

1. Please ensure that your manuscript meets PLOS ONE's style requirements, including those for file naming. The PLOS ONE style templates can be found at
   https://journals.plos.org/plosone/s/file?id=wjVg/PLOSOne_formatting_sample_main_body.pdf and  https://journals.plos.org/plosone/s/file?id=ba62/PLOSOne_formatting_sample_title_authors_affiliations.pdf

   **Reply AS:** Thank you. Now, all modifications were implemented in accordance with the style criteria of PLOS ONE.
2. We note that the grant information you provided in the ‘Funding Information’ and ‘Financial Disclosure’ sections do not match.
   When you resubmit, please ensure that you provide the correct grant numbers for the awards you received for your study in the ‘Funding Information’ section.

**Reply AS:** Yes, you are right. Please excuse the inconsistencies regarding the financial disclosure. As stated in the provided financial disclosure statement: “The University Medicine Greifswald and Zentralklinik Bad Berka have received non-targeted financial support by Redwave Medical GmbH for carrying out the study. The main parts of the study were financed by the internal funds of the participating hospitals. Redwave Medical GmbH had no role in study design, data collection and analysis, decision to publish, or preparation of the manuscript. The authors received no specific funding for this work. The publication of this article was funded by the Open Access Publishing Fund of Leipzig University supported by the German Research Foundation within the program Open Access Publication Funding.”.

We’ve also added the financial disclosure statement to the cover letter.

**Reviewer’s comments to the Author**

**Reviewer #1**

In this very interesting article, the authors evaluated the agreement/correlation of the Antares oscillometric pulse wave analysis algorithm and Doppler transthoracic echocardiography (TTEcho) for cardiac output measurements in patients undergoing elective cardiac catheterization for clinical reasons. The authors demonstrate that Antares oscillometric pulse wave analysis algorithm measurements are comparable to measurements by TTEcho.

1. Although the authors observed a strong correlation and a good agreement between the methods, the Antares oscillometric pulse wave analysis algorithm could not determine cardiac output in 34% of cases due to inappropriate data. Can the authors explain why the algorithm could not determine cardiac output in these patients? Could this limit the feasibility of using the algorithm as an alternative to TTEcho in any clinical setting?

**Reply AS:** Good point. Thank you very much for your input. In chapter “Cardiac output estimation with the Antares algorithm” (lines 89-97), we have presented the information provided by Redwave Medical regarding the criteria for determining CO, emphasizing the necessity for robust grid point detection and automatic exclusion of pulse waves if these criteria are not met. Due to Redwave Medical's intellectual property protection, we are unable to provide further details. Nevertheless, it remains unclear why the corresponding grid points were not distinctly identifiable and/or stable for pulse wave analysis in these patients. Possible factors could include signal disturbances or certain pulse wave morphologies (see Fig. 1B) where necessary clear differentiation between the first and second shoulders of the systolic pressure and the dicrotic notch is not evident within the signal.

However, the inability of the algorithm to determine CO in all cases at present restricts its applicability as an alternative to TTE in clinical settings. We have incorporated this information into the limitations section of the discussion and hope that you agree with our proceed.

2. The authors should specify the sampling method and how the sample size was defined in the Methods.

**Reply AS:** Thank you also for this suggestion for providing details on sampling method and sample size. Since cardiac output was not a primary (central blood pressure and PWV) or secondary target criterion (clinical validation) of the multicenter study, there was no specific sample size planning for this parameter. For the sub-study conducted between February 2019 and September 2020, a total of 90 patients with complete cardiac output data (stroke volume, heart rate, left ventricular ejection fraction) were identified from medical records at the Bad Berka study center, and these data were utilized for the current study. Study participation, based on inclusion/exclusion criteria, was independent of underlying disease/condition, as the only inclusion criteria were clinical indication for cardiac catheterization, age ≥ 18 years, and ability to provide informed consent. This information was detailed under "Study population" in the "Materials and methods" chapter. Additionally, details regarding ethnicity and age distribution were incorporated now.

#3 - The reasons for the elective cardiac catheterization should be present in the Results.

**Reply AS:** Thank you for suggesting the inclusion of specific reasons for elective cardiac catheterization. Regrettably, the authors currently lack data on the indications, and obtaining this information would necessitate another extensive review of medical records by hospital staff. However, if this aspect is deemed necessary for formal acceptance of the manuscript, we are prepared to coordinate with the clinic to revisit the data accordingly.

#4 - In the Results, the authors stated, "Regarding the hemodynamic data collected using TTE, cardiac catheterization (aortic BP), and the oscillometric brachial BP device, the statistical analysis showed no statistically significant differences among the entire group, female, and male patients, or between the sexes, except for a significantly higher aortic diastolic BP observed in the female group (Table 2)". However, the LVEF was lower in males than females (p = 0.035).

**Reply AS:** Thank you for bringing this error to our attention. We have rectified it accordingly. Thank you very much!

#5 - In the Discussion, the authors should mention the inherent limitations of the Antares oscillometric pulse wave analysis algorithm and TTEcho to ensure readers understand the methods' limitations.

**Reply AS:** Agreed and thank you for your feedback. We have incorporated further significant limitations of both methods into the discussion to provide a clearer understanding for the readers.

#6 - Is it possible to explain the best correlations between the Antares oscillometric pulse wave analysis algorithm in men rather than women?

**Reply AS:** A further good point. The observed differences in correlation between female (r=0.80) and male (r=0.67) patients could be ascribed to various factors. These encompass variances in vascular function, differences in heart size and function, hormonal influences on cardiovascular dynamics, disparities in body composition (affecting pressure wave transmission), potential discrepancies in underlying cardiovascular diseases between sexes, and the possibility of sampling bias. Further research is needed to gain a deeper understanding of these sex-specific differences and the underlying mechanisms, with the future aim of developing an algorithm that provide comparable performance in both sexes. We have now incorporated this important aspect into our discussion. At this point, we would like to thank you again for your time and effort for providing us with constructive feedback that has help us to improve our paper.
